# Application of Plant Extracts to Control Postharvest Gray Mold and Susceptibility of Apple Fruits to *B. cinerea* from Different Plant Hosts

**DOI:** 10.3390/foods9101430

**Published:** 2020-10-09

**Authors:** Lina Šernaitė, Neringa Rasiukevičiūtė, Alma Valiuškaitė

**Affiliations:** Laboratory of Plant Protection, Institute of Horticulture, Lithuanian Research Centre for Agriculture and Forestry, Kauno st. 30, LT-54333 Babtai, Kaunas dist., Lithuania; neringa.rasiukeviciute@lammc.lt (N.R.); alma.valiuskaite@lammc.lt (A.V.)

**Keywords:** antifungal activity, biocontrol, *Botrytis cinerea*, postharvest, preservation, susceptibility

## Abstract

Sustainable plant protection can be applied on apples against fungal pathogens such as *Botrytis cinerea* (which is responsible for gray mold)—a significant global postharvest disease. This pathogen can affect a wide range of hosts; and fruits may have variable susceptibilities to *B. cinerea* from different plant hosts. New possibilities to control gray mold in food production are under demand due to the emergence of resistance against antifungal agents in fungal pathogens. Cinnamon, pimento, and laurel extracts were previously assessed for antifungal activities under in vitro conditions and were found to have the potential to be effective against postharvest gray mold. Therefore, this study aimed to investigate the antifungal activity of cinnamon, pimento, and laurel extracts in vitro and against postharvest gray mold on apples to determine the susceptibility of apple fruits to *B. cinerea* from different plant hosts, and to analyze the chemical composition of the extracts. Apples (cv. “Connell Red”) were treated with different concentrations of extracts and inoculated with *B. cinerea* isolates from apple and strawberry followed by evaluation of in vitro antifungal activity. The results reveal that most of the concentrations of the extracts that were investigated were not efficient enough when assessed in the postharvest assay, despite having demonstrated a high in vitro antifungal effect. Apples were less susceptible to *B. cinerea* isolated from strawberry. To conclude, cinnamon extract was found to be the most effective against apple gray mold; however, higher concentrations of the extracts are required for the efficient inhibition of *B. cinerea* in fruits during storage.

## 1. Introduction

Heavy losses in economically important horticultural plant yield are usually caused by *Botrytis* spp., and mainly *Botrytis cinerea*, which has an unlimited host range, various attack modes, and can survive in unfavorable conditions [1,2]. Necrotrophic *B. cinerea* infects and grows on damaged and senescing tissues, causing tissue death while exuding toxins and enzymes involved in reactive oxygen species [3]. The cost of damage caused by *Botrytis* spp. is in the range of tens to hundreds of billion dollars worldwide [2], because of its ability to affect all stages of production and become more resistant to existing fungicides [4,5]. Additionally, changes in the environment induce rapid mutations and the appearance of new strains, which are a threat to global food security [6]. Elevated temperatures and humidity in some ecozones cause increased growth and sporulation of *Botrytis* spp. [7,8].

*Botrytis cinerea* affects more than 200 plant hosts, including apple (*Malus domestica*) and strawberry (*Fragaria* × *ananassa*), and is widely spread in various regions [9,10]. This pathogen is highly aggressive due to its ability to mutate and create new strains. Choquer et al. [11] suggest that *B. cinerea* populations have developed strain-dependent infection strategies and have fractional but efficient adaptation to their different host plants. Populations of *B. cinerea* are more likely to exchange genes with an isolate from the same host than from another [12]; however, differences could be found between strains infecting the same host [13]. Horticultural diseases may spread between plant species through the environment. Cultivars that are less susceptible to gray mold are chosen for industrial horticulture to avoid diseases. However, the susceptibility to gray mold (*B. cinerea*) from different hosts has not been widely investigated.

Postharvest horticultural plant protection usually relies on preharvest chemical control of early inoculation density, but postharvest diseases caused by *Botrytis* spp. remain a common problem in storage [14]. Thiabendazole, phenylpyrrole, benomyl, and fludioxonil are widely used for gray mold control [15]. However, the study of Munoz et al. [16] revealed that four to five classes of fungicides have become less effective at *B. cinerea* inhibition. Although synthetic fungicides remain the main tool in pathogen maintenance because of their favorable price and effective usage, integrated disease management (IDM) is required for *B. cinerea* disease control [17,18]. Valiuškaitė et al. [19] demonstrated that a sustainable plant protection system, where low-toxicity products are not used more than twice, can help to maintain higher apple fruit quality. Sustainable plant protection can be useful against fungal pathogens on apples, such as in the case of gray mold caused by *B. cinerea*, which represents a significant disease for apples in storage worldwide. Jurick et al. [20] stated that *B. cinerea* isolates from apples have mutations in genes related to chemical resistance and may be developing additional mechanisms for protection. Despite the increased resistance of the pathogens, chemical fungicides have harmful compositions and result in an accumulation of side metabolites and other active substances in products and adverse effects for the environment and consumers [21]. Due to pathogen resistance to fungicides, plant disease management becomes more complicated and, therefore, alternative plant protection strategies are currently under investigation.

Biological agents and chemical compounds from plants are increasingly used for plant protection [22]. Many types of plant essential oils have the potential to preserve food products [23]. Therefore, the number of studies on the application of natural extracts in the food safety field is increasing, including the investigation of plant oils for inhibition activity in the context of postharvest gray mold caused by *B. cinerea*. The aromatic plants *Cinnamomum cassia* (cinnamon), *Pimenta dioica* (pimento), and *Laurus nobilis* (laurel) and their oil and extract components are known for their antibacterial properties and their richness in antioxidants and other active substances [24,25,26,27,28]. Studies have shown that cinnamon oil can inhibit some bacterial strains isolated from food [29] and fungal pathogen growth [30,31]. It was also investigated for its antimicrobial and antibiofilm activities [32], and as a potential biopreservative [33]. Cinnamon oil and its components express their antimicrobial activity by affecting various pathogen cell structures [34]. Extracts from pimento also demonstrated the presence of bioactive agents that can inhibit pathogenic bacteria [35], gray mold on strawberry leaves [36], and the mycotoxin producer *Aspergillus flavus* [37]. Antifungal activities of laurel oil were evaluated against spoilage fungi [38,39]. Laurel oil was studied for controlling bean rust and *Aspergillus* spp. [40], *B. cinerea* from strawberry in vitro [41], and *Salmonella* Typhimurium and *Escherichia coli* on fresh produce [42]. In reviewing the literature, attempts to control gray mold on plants and fruits with plant essential oils and extracts could also be found. Thyme, mint, and rosemary essential oils showed moderate effectiveness in *B. cinerea* management on vineyards [43]. Lemongrass and citrus can also be used for postharvest apple protection from *B. cinerea* [44]. Daniel et al. [45] studied the antifungal activity of garlic extracts and clove oil and revealed their effectiveness against gray mold on apples. The application of neem extracts on apple fruits also showed positive results for gray mold inhibition [15]. Meanwhile, Banani et al. [46] determined that thyme and savory essential oils are effective against *B. cinerea* on apple fruits. In other fruit tests, cinnamon oil completely controlled *A. flavus*, *Penicillium expansum*, and *Rhizopus nigricans* on wound-inoculated jujube and orange fruits at concentrations of 2.0% and 3.0% [30]. Some plants used in the food industry can be a target for antifungal activity studies, not only to investigate the potential of various plant essential oils and extracts but also to determine efficient concentrations.

Cinnamon, pimento, and laurel are used in the food industry and their essential oils and extracts have been investigated for potential use against bacterial and fungal pathogens for horticultural postharvest protection. Apples are common, widely produced fruits, and their storage protection is relevant in various regions, with *B. cinerea* remaining one of the most harmful postharvest pathogens. Thus, this study aimed to investigate the antifungal activity of cinnamon, pimento, and laurel extracts in vitro and against postharvest gray mold (*B. cinerea*) on apples, to determine the susceptibility of apple fruits to *B. cinerea* from different plant hosts after application of plant extracts, and to analyze the chemical composition of the extracts.

## 2. Materials and Methods

### 2.1. Fungal Material and Apple Fruits

The *B. cinerea* strains used in the experiments were isolated from rotten apples and strawberries by placing infected tissues onto solid PDA (potato dextrose agar) medium and transferring fungal mycelium onto a new PDA plate. Finally, a single spore (obtained by the dilution method) of each strain was transferred to fresh PDA and prepared for identification, which was performed in a previous study [47]. Isolates were renewed on PDA, incubated at 22 °C in the dark, and kept refrigerated at 4 °C until the beginning of the experiments.

Apple fruits (cv. “Connell Red”) were harvested in September 2019 from the orchards of the Institute of Horticulture (IH), Lithuanian Research Centre for Agriculture and Forestry (LAMMC) and kept at 6 °C in storage. Cultivar “Connell Red” is moderately susceptible to gray mold and was chosen to register the differences between infections caused by two pathogen isolates of different plant host origins.

### 2.2. Antifungal Activity In Vitro

The research took place at the Laboratory of Plant Protection, IH, LAMMC. The concentrations of extracts used for the experiment were 2200, 2400, and 2600 μL/L pimento; 400, 600, and 800 μL/L cinnamon; and 2600, 2800, and 3000 μL/L laurel. The corresponding amount of each extract was mixed with sterilized, cooled PDA medium, and poured into sterile Petri dishes. Then, a 7 mm plug of 7-day old *B. cinerea* isolated from apple and strawberry (from the margin of the plate) was placed in the center of each Petri dish, mycelium side down. Control plates contained PDA without extract. Radial colony growth (cm) was registered three times: 2, 4, and 7 days after inoculation (DAI) during incubation at 22 °C temperature in the dark. Extract was considered to have 100% antifungal effect if there was no visible pathogen growth after 7 days.

### 2.3. Antifungal Activity of the Extracts against Postharvest Gray Mold

Apples of the same maturity and without disease symptoms, as assessed visually, were collected for further assays to determine the antifungal activity of extracts on apple fruits. Fruits were surface sterilized using 70% ethanol, rinsed with sterile water three times, and left to dry naturally in a sterile room. After drying, wounds (ø 7 mm, 5 mm depth) were made vertically in the center of each apple on opposite sides using a sterile tool. Spraying mixtures (treatments) were prepared using extracts and sterile distilled water at the following concentrations: 2200 and 2600 μL/L pimento; 400 and 800 μL/L cinnamon; and 2600 and 3000 μL/L bay laurel. Each mixture (5 mL) was sprayed on 6 apples and left to dry for 30 min in a sterile room. Later, 7-mm plugs of *B. cinerea* isolated from apple and strawberry were placed in the wounds. Control apples were sprayed only with sterile distilled water. Apples were placed in ziplock plastic bags incubated at 25 °C temperature. The experiment was carried out with four replicates (six apples per replicate, each apple with two wounds). Lesion diameter (mm) was measured at 7 and 9 DAI. Antifungal activity was calculated as mycelial growth inhibition using the formula adopted by Mbili et al. [44]: mycelial growth inhibition (%) = (dc − dt)/dc × 100, where dc is lesion diameter in the control (mm) and dt is lesion diameter in the treated samples (mm).

### 2.4. Susceptibility of Apple Fruits to B. cinerea

To evaluate the susceptibility of apple fruits to different plant host isolates of *B. cinerea*, the number of infected lesions caused by strawberry *B. cinerea* and apple *B. cinerea* on apples treated with extracts was counted at 7 and 9 DAI, and disease incidence was calculated using the formula adjusted based on Youssef and Roberto [48]: disease incidence = (number of infected lesions/total number of assessed lesions) × 100.

### 2.5. Plant Extracts and Their Composition

Extracts used in the study were produced from dried pimento fruits (*Pimenta dioica*), cinnamon bark (*Cinnamomum cassia*), and laurel leaves (*Laurus nobilis*) using the method of CO_2_ extraction, as described in a previous study [41].

Gas chromatography–mass spectrometry (GC–MS) was used to determine the volatile components of produced extracts under previously specified conditions [49].

### 2.6. Statistical Analysis

Statistical analysis was performed with SAS Enterprise Guide 7.1 program (SAS Inc., Cary, NC, USA). Data obtained in experiments were analyzed using the analysis of variance (ANOVA) procedure. Treatment means were separated at a 5% significance level using Duncan’s multiple range test.

## 3. Results

### 3.1. Antifungal Activity of the Extracts In Vitro

**Figure 1:**
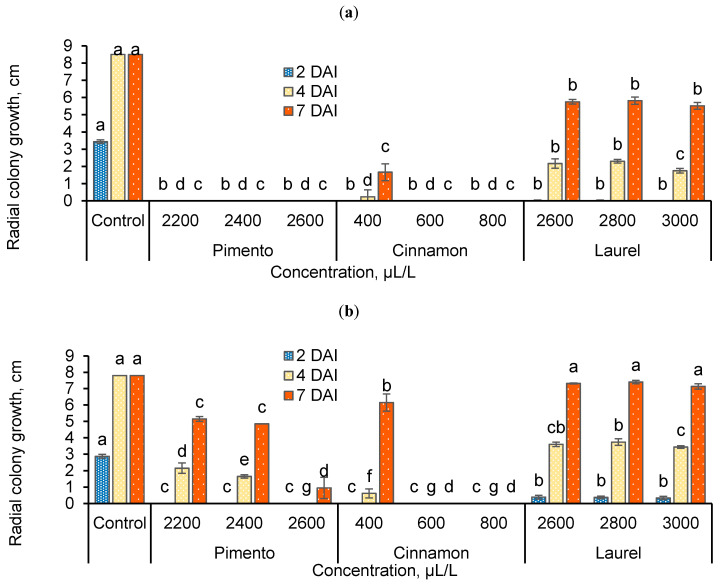
Radial colony growth (cm) on potato dextrose agar medium with different concentrations of extracts for *Botrytis cinerea* isolated from (**a**) strawberry and (**b**) apple. DAI—days after inoculation; the same letters indicate no significant differences between treatments according to Duncan’s multiple range test (*p* < 0.05) at 2, 4, and 7 DAI.

Radial colony growth of the apple and strawberry pathogen *Botrytis cinerea* during the experimental period is presented in Figure 1. Most of the concentrations of plant extracts suppressed the growth of both *B. cinerea* varieties at 2 DAI. The cinnamon and pimento extracts showed the best antifungal activity against strawberry *B. cinerea* in vitro. No significant differences were observed between 2200, 2400, and 2600 µL/L pimento extract and 600 and 800 µL/L cinnamon extract at 2, 4, and 7 DAI. The pathogen treated with different concentrations had no visible growth during the observation period (except cinnamon extract at 400 µL/L, which had low growth) and had significant differences from the control at each DAI. Meanwhile, pimento extract had a moderate antifungal effect against apple *B. cinerea* at 2200 and 2400 µL/L and cinnamon extracts were also less effective at 400 µL/L. Laurel extract had similar low antifungal activity against both isolates of *B. cinerea*. Significant differences from other treatments, except the control were observed at each DAI.

### 3.2. Antifungal Activity of the Extracts against Postharvest B. cinerea

**Figure 2:**
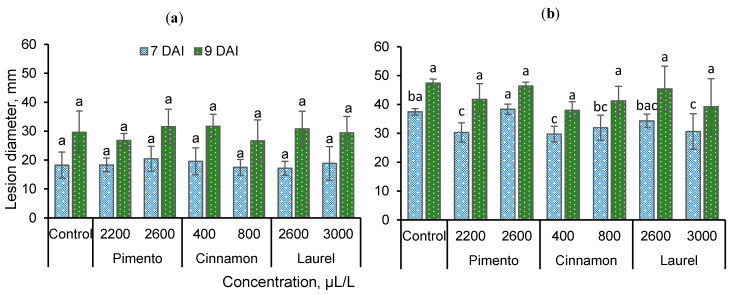
Lesion diameter (mm) on apple fruits after treatment with different concentrations of extracts: (**a**) rot caused by strawberry *Botrytis cinerea*; (**b**) rot caused by apple *B. cinerea*. DAI—days after inoculation; the same letters indicate no significant differences between treatments according to Duncan’s multiple range test (*p* < 0.05) at 2, 4, and 7 DAI.

The postharvest antifungal activity of the extracts is shown in Figure 2. Lesions caused by strawberry *B. cinerea* did not significantly different in the control apples without any extract compared to the apple fruits with applied extracts (Figure 2a) at 7 DAI. Treatment with 2200 µL/L pimento extract and 800 µL/L cinnamon extract resulted in the smallest lesions at 9 DAI. Treatment with 2200 µL/L pimento extract, 400 µL/L and 800 µL/L cinnamon extract, and 3000 µL/L laurel extract significantly reduced lesions caused by apple *B. cinerea* compared with the control at 7 DAI (Figure 2b). The smallest lesion at 9 DAI was observed on 800 µL/L cinnamon extract-treated apples.

Mycelial growth inhibition was determined according to the diameters of the lesions for different treatments (Table 1). The results demonstrate that extracts showed low postharvest antifungal activity on apples inoculated with less aggressive strawberry *B. cinerea* compared to the control. Some of the extracts even increased the spread of the lesions (negative mycelial growth inhibition observed). By contrast, application of cinnamon extract showed the highest inhibition of apple *B. cinerea* compared to the control.

### 3.3. Susceptibility of Apple Fruits to B. cinerea

Disease incidence on apples treated with extracts at 7 days after inoculation is presented in Table 2. It was observed that apples with application of 800 µL/L cinnamon oil had the least strawberry *B. cinerea*-infected lesions, while treatments with 2200 µL/L pimento and 2600 µL/L laurel also reduced the susceptibility of apples. Other treatments had no influence. Fruits were found to be more susceptible to apple *B. cinerea* as all treated apples had infected lesions at 7 DAI except those treated with 2600 µL/L pimento. That treatment had lower disease incidence than others. No differences between treatments were observed at 9 DAI. Overall, apple fruits were less susceptible to strawberry *B. cinerea* than apple *B. cinerea* as the lesions were spreading slower and not that widely in comparison after inoculation with the strawberry pathogen (Figure 2).

### 3.4. Composition of Plant Extracts

The results of gas chromatography–mass spectrometry of volatile components of the investigated extracts are presented in Table 3. The main component of pimento (*P. dioica*) extract was methyleugenol. A high quantity of eucalyptol and eugenol were also found in this extract. Meanwhile, laurel (*L. nobilis*) extract mainly contained eucalyptol and α-terpinyl acetate. A total of 99.50% and 87.99% of volatiles were identified in the pimento and laurel extracts, respectively. Information about the cinnamon (*C. cassia*) extract used in the experiments can be found in a previous study [49].

## 4. Discussion

The main active volatile components of the extracts used in this study were pimento—methyleugenol (44.39%), eucalyptol (11.81%), and eugenol (10.72%); laurel—eucalyptol (29.10%) and α-terpinyl acetate (18.25%); cinnamon—*trans*-cinnamaldehyde (74.67%). The obtained cinnamon and laurel extract compositions were in agreement with earlier research [23,31,38,39]; however, there are other dominating components found in the pimento extract here, which could depend on the plant parts used for extraction and the parameters of the raw material. The influence of *trans*-cinnamaldehyde and eugenol, and their derivatives, on the antifungal properties of oils and extracts has been previously reported [26,27,28]. The antimicrobial activity of the essential oils of *Eucalyptus* plants and the main component eucalyptol (1,8-cineole) was compared in the study of Safaei-Ghomi and Ahd [24]. The authors concluded that oils were more effective than the main component alone due to the existence of other volatile components in the whole oil. The same conclusions were reached by Dammak et al. [39] about *L. nobilis* essential oil and eucalyptol. Another study [33] showed cinnamon oil at concentrations close to what we used for *trans*-cinnamaldehyde (63.58%) was an effective antimicrobial agent for wine preservation. According to the results of this study and data found in the literature, the composition of the investigated extracts is promising in terms of antifungal properties.

Eugenol was previously determined to be highly active against Fusarium subglutinans, F. cerealis, F. verticillioides, F. proliferatum, F. oxysporum, F. sporotrichioides, Aspergillus tubingensis, A. carbonarius, Alternaria alternata, and Penicillium spp.; meanwhile, eucalyptol showed a mild effect [26]. Cinnamon oil (C. zeylanicum) inhibited the growth of F. oxysporum (antifungal index 54.8%) and Rhizoctonia solani (antifungal index 28.5%) at 400 µg/mL [27], and A. ochraceus [31]. Additionally, cinnamon bark oil and cinnamon oil completely inhibited mycelial growth of Villosiclava virens at 10 µL/L in air during a fumigation activity assay and 66.1% and 71.9% in the contact activity assay [23]. Moreover, they demonstrated effectiveness in inhibition of conidial germination. Our results are in agreement with those of other studies as cinnamon extract (74.67% *trans*-cinnamaldehyde) was highly active against two isolates of B. cinerea, though at significantly lower concentrations. The literature on laurel oil is characterized by diverse information regarding its antifungal properties. Laurel essential oil had high antifungal activity against A. carbonarius (minimal inhibitory concentration (MIC) (0.3%) while its constituent eucalyptol was less effective and had higher MIC (0.5%) [39]. Corato et al. [38] found laurel essential oil to be highly effective against Monilinia laxa at 200 µg/mL, B. cinerea at 1000 µg/mL, and partially effective against Penicillium digitatum. Antifungal activity against strains of Candida parapsilosis, C. gattii, and C. neoformans was confirmed [25]. Laurel essential oil had the lowest antimicrobial activities among other oils investigated in Rafiq et al. [42], and a similar result was obtained in our study. In the present study, cinnamon and pimento extracts (CO_2_-extracted) demonstrated high in vitro antifungal activity against two different isolates of B. cinerea from strawberry and apple fruits, and laurel extract had the lowest. In the aforementioned studies by other authors, significantly higher concentrations of this oil were investigated; consequently, an increase in the concentration range is required for an effect against B. cinerea as seen in our results. Pimento oil showed 73.95–100% antifungal activity at 1.5–2.5 µL/mL against A. flavus [37]. Results of our in vitro tests revealed high antifungal activity of pimento extract with three dominant compounds at comparable concentrations, which may be the result of synergy between components [24,39]. To conclude, plant extracts demonstrate various antifungal activities against fungal pathogens that depend on the composition, which may vary due to the extraction process, raw material parameters, and fungal pathogen tested. Thus, the standardization of extracts should be required for the determination of antifungal concentration range. As different antifungal effects against B. cinerea from two plant hosts were observed, our recommendations would be to highlight the origin of fungal isolates of the same genus in the research because varied results may be obtained according to this factor.

During further analysis, plant extracts were investigated for antifungal activity against postharvest *B. cinerea* on “Connell Red” apples. No significant influence of treatment with extracts on the apple gray mold infection caused by strawberry *B. cinerea* was observed. However, for each extract, one of the tested concentrations was found to mildly suppress the infection induced by *B. cinerea* isolated from apple. Contrarily, various plant methanol extracts (15–25%) showed the possibility to control gray mold on “Golden Delicious” apples [15]. Meanwhile, 40% and 50% garlic extracts and 1% clove oil showed promising results by strongly inhibiting *B. cinerea* on the apple cultivars “Granny Smith”, “Golden Delicious”, and “Pink Lady” at 7 DAI [45]. Similarly, lemongrass and citrus oils were effective against *B. cinerea* on “Golden Delicious”, “Pink Lady”, and “Granny Smith” apple cultivars [44]. The authors of this study also stated that the differences between the results of in vitro effectiveness of extracts and the effect in vivo may depend on interactions between host tissues, the pathogen, and the environmental impact on physiology and metabolism of both the host and the pathogen. We agree with this statement as the concentrations of our investigated extracts with 100% in vitro antifungal activity had a mild antifungal effect on apple fruits. Apples in our study were protectively treated with extracts (before inoculation); however, not all tested concentrations were effective at 7 DAI. Concentrations equal to the in vitro concentrations resulted in mild antifungal activity when tested on apple fruit; however, the results provide information for possible future tests in agreement with the fact that the higher concentrations of oils in the other mentioned studies were able to control *B. cinerea* on apples. Successful control of *B. cinerea* in apples with 1% thyme oil was related to the induction of defense mechanisms in fruits [46].

Preharvest conditions during cultivation are responsible for the accumulation of phytochemicals in apples, which then influences postharvest resistance to pathogens [10]. In this study, fruits were inoculated with pathogens of apple and strawberry origin to investigate the susceptibility of apples to *B. cinerea* from different plant hosts. “Connell Red” apples were found to be more susceptible to apple *B. cinerea* than strawberry *B. cinerea*, which confirms the adaptation of *B. cinerea* to their plant host [11]. Similar results were obtained with tomato *B. cinerea* which was found to germinate faster on tomato leaves than grape leaves [13].

## 5. Conclusions

Alternative methods for postharvest control of the harmful and aggressive pathogen *Botrytis cinerea* are currently under investigation. In this study, “Connell Red” apples were less susceptible to strawberry *B. cinerea* than the apple variety, and the differences between the isolates from the two plant hosts were confirmed. Despite the demonstrated high in vitro antifungal activity, the investigated extracts were not equally effective when applied on apple fruits. In summary, cinnamon extract had the best antifungal activity against apple *B. cinerea* in fruits of those tested in our study; however, no significant effect of any of the extracts was observed on the inhibition of strawberry *B. cinerea*. The research on the tested extracts should be continued and include an evaluation of the sensory properties of the fruits. While low concentrations of the aromatic plant extracts might not have a high impact on the odor and taste of apples, the higher concentrations that appear to be required for the inhibition of *B. cinerea* in fruits during storage may have a noticeable effect on fruit properties.

## Figures and Tables

**Table 1:** Mycelial growth inhibition of *B. cinerea* on apples by different extracts compared to the control at 7 days after inoculation.

Extract	Concentration, µL/L	Inhibition of Strawberry *B. cinerea*, %	Inhibition of Apple *B. cinerea*, %
Pimento	2200	−0.55	19.09
	2600	−12.21	−2.36
Cinnamon	400	−7.41	20.63
	800	3.98	14.69
Laurel	2600	5.76	8.41
	3000	−3.43	18.22

**Table 2:** Disease incidence on apples treated with extracts at 7 days after inoculation.

Extract	Concentration, µL/L	Inoculated with Strawberry *B. cinerea*, %	Inoculated with Apple *B. cinerea*, %
Pimento	2200	91.67	100
	2600	100	83.33
Cinnamon	400	100	100
	800	83.33	100
Laurel	2600	91.67	100
	3000	100	100

**Table 3:** Volatile components of pimento (*P. dioica*) and laurel (*L. nobilis*) extracts. Results are presented as means (*n* = 3).

	*Pimenta dioica*		*Laurus nobilis*	
Component	PA ^1^ (%)	RT ^2^	PA (%)	RT
α-Pinene	0.69	6.694	1.04	6.668
Sabinene	1.45	7.718	2.10	7.692
β-Pinene	0.73	7.810	1.24	7.782
Myrcene	5.13	8.185		
*p*-Cymene	0.28	9.122	0.56	9.091
Limonene	0.35	9.253	0.37	9.197
Eucalyptol	11.81	9.346	29.10	9.328
*cis*-Sabinene hydrate			0.20	10.335
*trans*-β-Ocimene	0.59	9.752		
Linalool	0.83	11.265	3.40	11.242
Delta-terpineol			0.35	13.192
Terpinen-4-ol	0.47	13.505	2.17	13.473
α-terpineol	0.58	13.905	1.54	13.867
Estragole	0.31	14.064		
Linalyl acetate			0.35	15.528
Trans-cinnamaldehyde			2.04	16.087
δ-Terpinyl acetate			1.21	17.236
α-Terpinyl acetate	0.91	18.139	18.25	18.158
Eugenol	10.72	18.546	3.25	18.394
α-Ylangene			0.24	18.721
β-Elemene	0.94	19.297	0.86	19.242
Methyleugenol	44.39	19.861	2.04	19.532
*trans*-Caryophyllene	6.67	20.114	1.97	19.983
1-Methyl-4-(1-acetoxy-1-methylethyl)-cyclol			0.38	20.279
6,9-Guaiadiene + coumarin			0.31	20.531
Isogermacrene D			0.22	20.686
α-Humulene	1.21	20.925	0.34	20.835
α-Neoclovene			0.37	20.891
Germacrene D	0.57	21.583		
β-selinene	0.24	21.721	0.31	21.650
*Trans*-methyl isoeugenol + viridiflorene	0.30	21.921		
γ-cadinene			0.34	22.300
Eugenyl acetate			0.47	22.524
Trans-α-bisabolene			0.74	22.904
Spathulenol			0.28	23.918
Caryophyllene oxide	0.48	24.108	0.84	24.042
Caryophylla-4(12),8(13)-dien-5-α-ol			0.33	25.586
β-Eudesmol			0.40	26.002
Neointermedeol			0.43	26.128
Dehydrosaussurea lactone			1.20	29.403
*m*-Camphorene	0.30	30.907		
Hexadecenoic acid	0.47	31.072		
*p*-Camphorene	0.42	31.328		
Methyl linoleate	0.26	32.468		
Phytol			0.38	32.674
Linoleic acid	3.26	33.025	1.95	32.985
Stearic acid	1.77	33.242		
Other ^3^	3.37		5.1	
Total identified	99.50%		87.99%	

^1^ PA—peak area. ^2^ RT—retention time. ^3^ Consists of components less than 0.2% of total extract.
